# Practical Points in Nursing

**Published:** 1911-01

**Authors:** 


**Practical Points in Nursing.** By **Emily A. M. Stoney.** Fourth edition. 12 mo, pp. 495. Illustrated. Philadelphia and London: W. B. Saunders Co. 1910. (Cloth, $1.75.)

The rapid exhaustion of several large editions of this work would certainly seem to recommend it as a book of great value to the nursing profession. A number of subjects of much interest are discussed in a plain and candid manner. Some very level-headed advice, is given on the duties and rights of nurses, which should make good reading and food for consideration on the part of many nurses now in the field. Medical, surgical, obstetrical, and gynecological cases are taken up and discussed as fully as is required. Accidents and emergencies are dealt with, and a chapter is given to the nursing of sick children. The tables, dose list, and glossary in the back of the book are useful. Every nurse should have a copy of this practical work. J. A. R.

